# Chyluria Associated With Nephrotic-Range Proteinuria

**DOI:** 10.7759/cureus.26690

**Published:** 2022-07-09

**Authors:** Amninder Kaur, Sharon Kandari, Sandeep Saini, Dipesh K Dhoot, Ashwani Kandari

**Affiliations:** 1 Nephrology, All India Institute of Medical Sciences Rishikesh, Rishikesh, IND; 2 Urology, All India Institute of Medical Sciences Rishikesh, Rishikesh, IND

**Keywords:** renal biopsy, nephrotic syndrome, filariasis, proteinuria, chyluria

## Abstract

Chyluria is the presence of chyle in the urine and is associated with some degree of proteinuria. We report two patients with chyluria who presented with milky urine, weight loss, and edema and were found to have nephrotic-range proteinuria. Although filarial antigen was detected in only one of the patients, flexible cystoscopy could demonstrate chyle efflux from the left ureter in first patient and from both the ureters in the second patient. Both patients received endoscopic sclerotherapy with 0.2% povidone-iodine, which resulted in the clearance of milky urine in three to five days and complete resolution of nephrotic-range proteinuria on follow-up. They remained symptom-free until the six-month follow-up. We deferred renal biopsy in both patients, as proteinuria was confirmed to be non-glomerular in origin.

## Introduction

Chyluria was described in 400 BC by Hippocrates. It was also named “suklameha” by Charak in 300 BC. It is defined as the excretion of chyle in urine, which is a milky fluid that contains chylomicrons and lymph. Chyle is taken up by intestinal lymphatic vessels and drained into the left subclavian vein via the thoracic duct. Normally, there is no communication between lymphatic vessels (lacteals) and the urinary tract. An abnormal communication between them causes the leakage of chyle into the urine. Lymphatic fluid predominantly contains albumin, leading to massive proteinuria. Herein, we report two cases of chyluria with nephrotic-range proteinuria, and their management resulted in the complete resolution of proteinuria.

## Case presentation

Case 1

A 56-year-old female resident of Uttar Pradesh, who had hypertension, was admitted with complaints of milky urine and weight loss for 1.5 months. She had no complaints of oliguria, dysuria, lithuria, fever, or flank pain. Her grandfather had filariasis.

**Figure 1:**
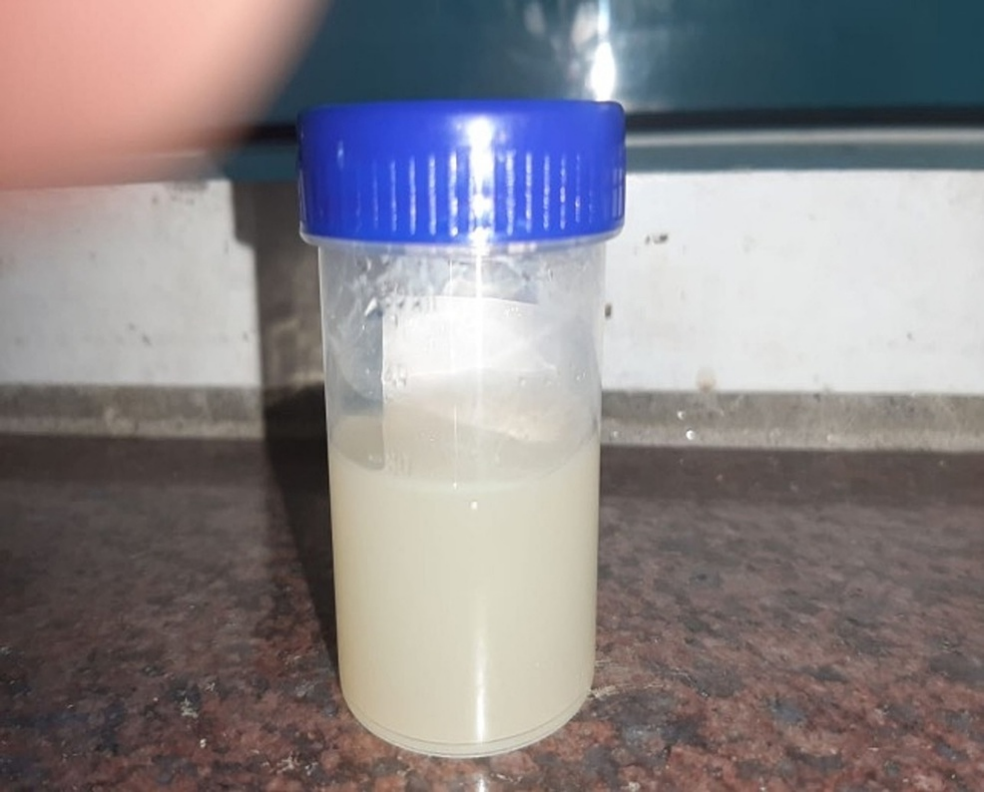
Milky urine (Case 1)

On admission, her blood pressure was 124/80 mmHg, and she had grade 1 bilateral pitting edema. Her body mass index was 20.2 kg/m^2^, and other results of the general, physical, and systemic examinations were unremarkable. She was taking a telmisartan tablet once a day. Macroscopically, her urine had a milky appearance (Figure 1). On ultrasonography, the kidney had a normal size, with no hydroureteronephrosis, calculi, or renal mass. Her urine was examined for malignant cells, and the acid-fast bacillus test was negative. Results of the rest of the investigations are presented in Table 1.

**Table 1:** Investigations on admission ICT, immunochromatographic card test; RBC, red blood cell; WBC, white blood cell

Investigations		Case 1	Case 2	Reference range
Hemoglobin (g/dL)		9.1	9.4	11.5–15.5
Peripheral blood smear		Microcytic, Hypochromic	Microcytic, Hypochromic	Normocytic, Normochromic
Serum creatinine (mg/dL)		0.59	0.4	0.59–1.04
Serum albumin (g/dL)		3.5	2.2	3.4–5.4
Urine Microscopy	Protein	2+	3+	Nil
	RBCs/hpf	10–13	40–50	0–5
	WBCs/hpf	10–12	0–1	0–4
Urine culture		Sterile	Sterile	
24-h urinary protein (/day)		4 gm	8.3 gm	<150 mg
Urinary triglyceride (mg/dL)		48	147	1–10
Serum triglyceride (mg/dL)		55	−	<150
Serum low-density lipoprotein (mg/dL)		74	−	<100
Serum filarial antigen by ICT		Negative	Positive	

Filarial immunoglobulin (Ig) M and IgG antibodies and serum filarial antigen (by immunochromatography) were negative. She was incidentally found to be hepatitis C virus (HCV) reactive, and its treatment was planned to be started later. The serum complement levels, including C3 and C4 levels, were within the normal range. Levels of tumor markers (carcinoembryonic antigen, cancer antigen 19.9, and cancer antigen 125) were within the normal range. Magnetic resonance cholangiopancreatography showed a distended gall bladder with cholelithiasis. Echocardiography revealed grade 1 diastolic dysfunction with concentric left ventricular hypertrophy, and the ejection fraction was normal (55%-60%).

**Figure 2:**
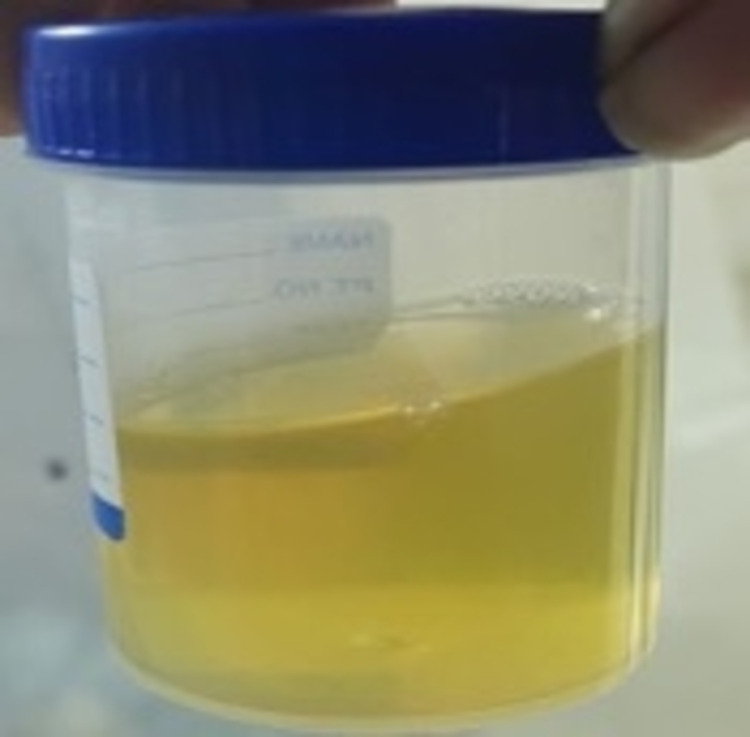
Urine on day 5 of sclerotherapy - Case 1

For further evaluation of chyluria, technetium-99m-sulfur colloid lymphoscintigraphy showed sluggish ascent of the tracer from the left inguinal nodes and cisterna chyli s/o left-sided thoracic duct obstruction. Diagnostic cystoscopy with retrograde pyelography revealed chyluria lateralizing to the left side, and retrograde pyelography showed the calyces (Figure 2).

**Figure 3:**
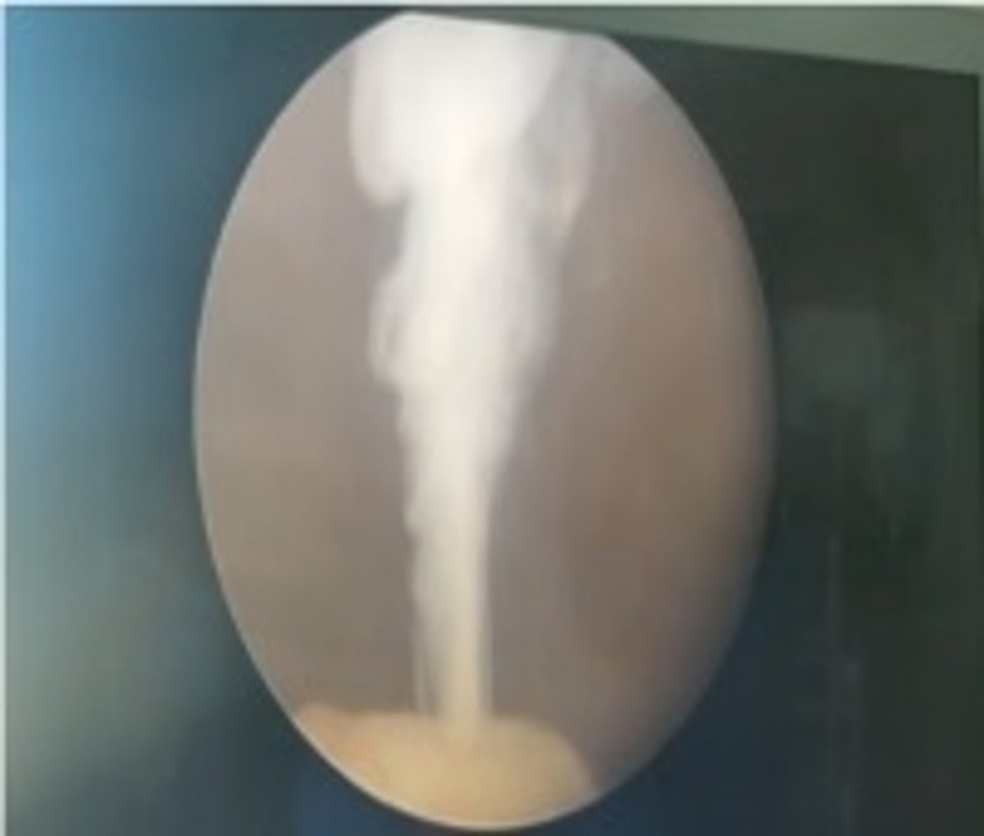
Cystoscopy demonstrating chyluria - Case 1

The patient was started on diethylcarbamazine empirically, as she had a positive family history. However, instillation therapy was started on day 10, as her symptoms persisted. Then, 7 mL of 0.2% povidone-iodine was instilled endoscopically to the left ureter via a 6-French ureteric catheter. The procedure was repeated twice a day for three consecutive days. She had an uneventful recovery, with complete clearance of urine over the next five days (Figure 3).

On routine follow-up after one month, urine was completely clear, with urine microscopy showing the absence of protein and red blood cells, and the 24-h urine protein was 40 mg/day, thus showing that the nephrotic-range proteinuria was chylous in origin. The patient was started with anti-HCV treatment after the resolution of chyluria and was followed up regularly for six months, with no recurrence of symptoms.

Case 2

**Figure 4:**
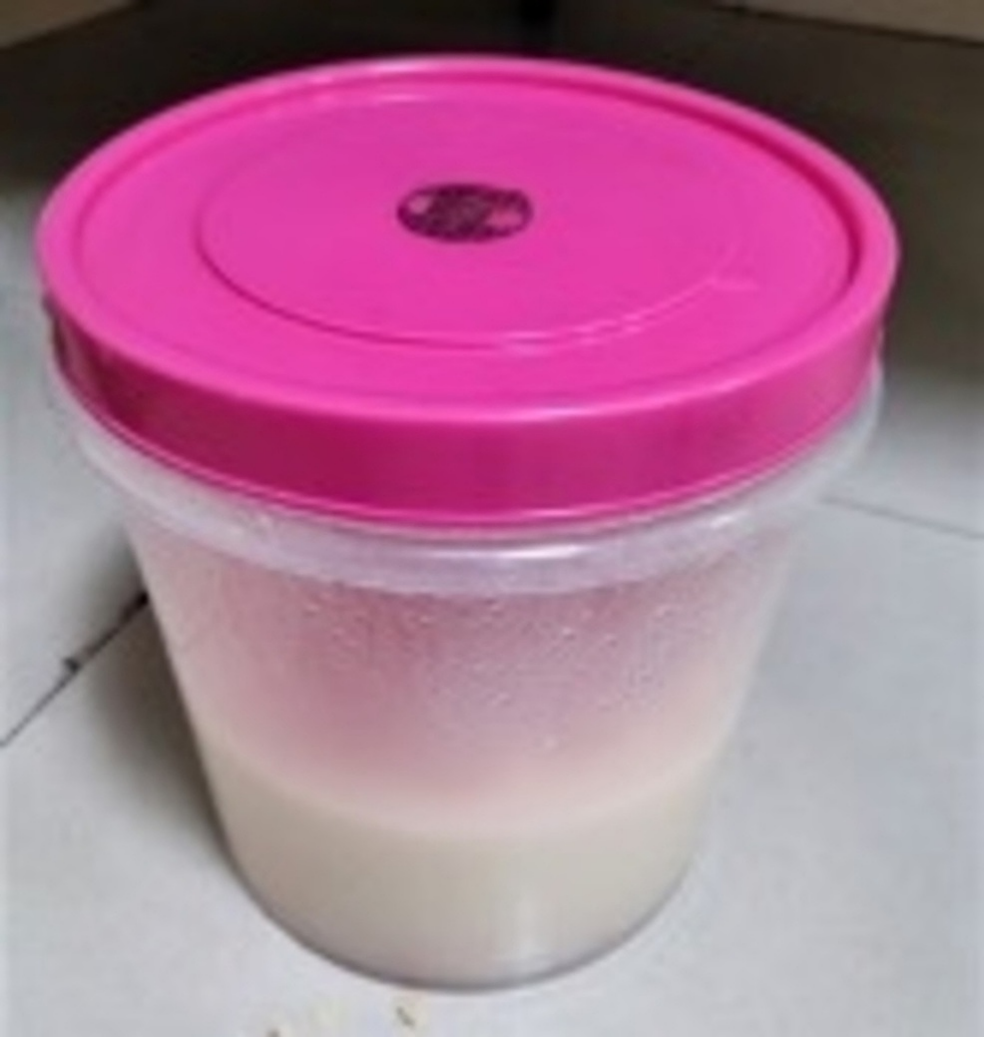
Milky urine - Case 2

A 21-year-old female resident of Uttar Pradesh presented with a history of passing white frothy urine with occasional reddish discoloration (Figure 4) around one week after full-term normal vaginal delivery. She had a history of a frequent passage of soft fleshy tissue in urine and colicky pain radiating from the loin to the groin on both sides occasionally. She had lost 8 kg of weight in the last two months. There was no history of limb swelling, facial puffiness, decreased urine output, or fever. She had no history of tuberculosis or any surgery. She was conscious, had normal vitals, and had no pedal edema. Her systemic examination was unremarkable. Laboratory results are shown in Table 1.

**Figure 5:**
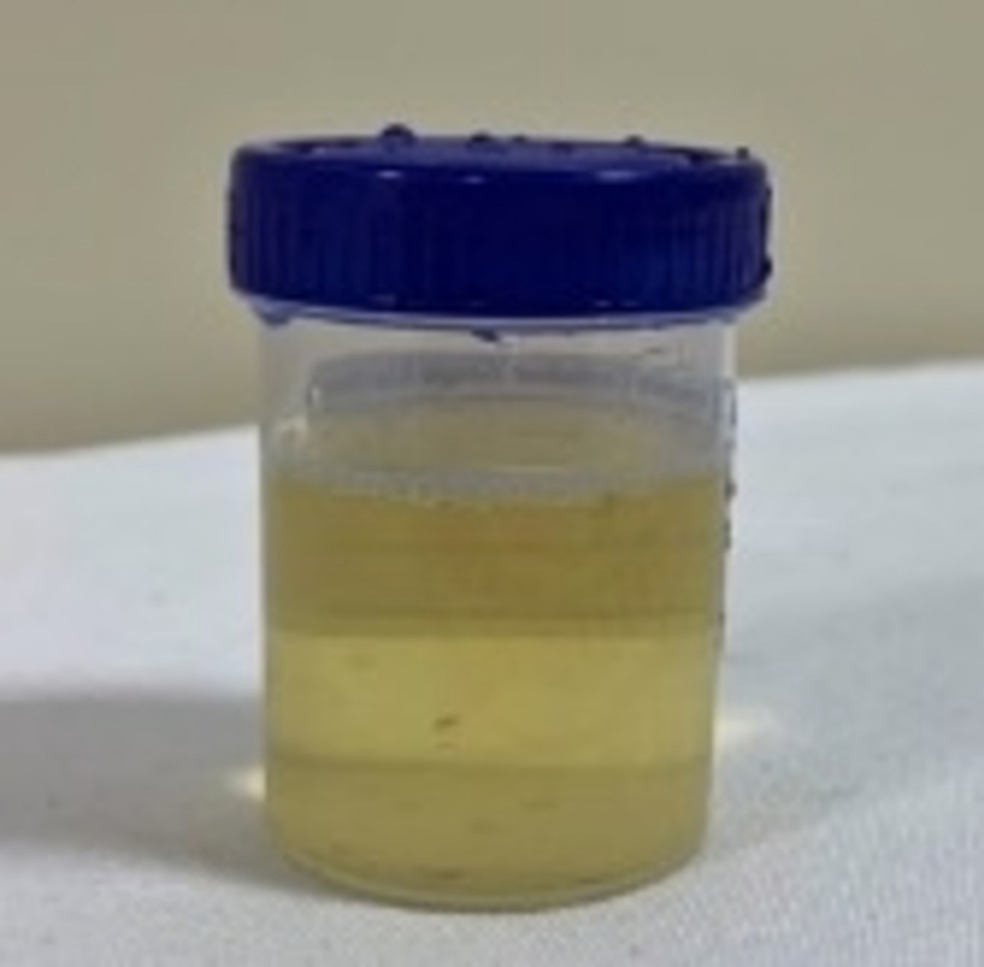
Urine on day 3 of sclerotherapy - Case 2

On abdominal ultrasonography, the kidney size was normal (right kidney = 9.7 cm, left kidney = 9.8 cm) with no hydroureteronephrosis, calculi, or renal mass. Cystoscopy revealed white efflux from both the ureteric orifices. Retrograde pyelography revealed mild chyluria from the right side and severe chyluria from the left side. The patient was started on daily instillation of 0.2% povidone-iodine solution in both pelvicalyceal systems through a ureteric catheter. A total of 10 doses were given. The patient had intermittent lower abdominal pain with dysuria, which was managed with analgesics. As her serum filarial antigen was positive, diethylcarbamazine 100 mg was given thrice daily for 12 days. Her chyluria and proteinuria were completely resolved on follow-up (Figure 5).

## Discussion

Chyluria is the presence of chyle in the urine. It is endemic in South Asia [1,2]. Chyluria indicates the presence of an abnormal communication between intestinal lymphatics and the urinary tract through a lymphaticourinary fistula. Causes are parasitic and non-parasitic. Wuchereria bancrofti is responsible for >90% of parasitic cases [3]. Up to 10% of patients living in endemic areas may be afflicted by filariasis, with chyluria occurring in only 2% of them [4].

Patients with chyluria present with milky or cloudy urine aggravated by a fatty diet. Chyluria is confirmed by urine examination. Sensitivity can be increased by urine collection 4 h after a fatty meal [5]. The presence of urinary triglycerides is 100% sensitive and specific [6]. Both patients had high urinary triglyceride levels and low serum lipid levels.

To diagnose filariasis, detection of microfilariae in a blood smear is a specific and inexpensive test, but it should be performed at night, hence uncomfortable. The immunochromatographic card test is more sensitive than a blood smear, but it cannot detect Brugia infections [7]. We identified the parasitic cause of chyluria in case 2 by immunochromatography. However, in case 1, we only had radiological evidence of left-sided thoracic duct obstruction.

Flexible cystoscopy confirms the presence of gelatinous urine and adipose fragments in the bladder and identifies the ureteral papilla, from which whitish urine is ejaculated. In both cases, chyluria was detected by flexible cystoscopy. Spontaneous remission occurs in 50% of the cases [8]. A high-protein and low-fat diet should be given in case of persistent chyluria and malnutrition [9]. Medical treatment includes antihelminthic drugs, such as diethylcarbamazine, ivermectin, and albendazole, for parasitic infection, angiotensin-converting enzyme inhibitors, and somatostatin analogs. Invasive approaches include endoscopic and surgical procedures. A sclerosing solution is most commonly used, and the technique has an 80% success rate [10]. Sclerosing agents, such as 0.1%-0.5% silver nitrate, 50% dextrose, 0.2%-5% povidone-iodine, 10%-25% potassium iodide, and 3% hypertonic saline have been used for sclerotherapy [11].

Surgery is indicated if the patient has refractory chyluria, especially those with systemic complications. Surgeries include nephrolympholysis, hilar stripping, ureterolympholysis, fasciectomy, and nephropexy [12]. Various management strategies have been employed for the management of chyluria. Tan et al. used only dietary modifications [3]. Saha et al. diagnosed chyluria by lymphoscintigraphy and found their patient to be positive for filariasis. They treated the patient with diethylcarbamazine initially; however, surgical closure was needed, as the patient did not respond to medical therapy [13]. Sivashankar et al. reported a case of chyluria that was managed by endoscopic sclerotherapy [14].

In case 1, the patient was initially started on medical therapy; however, sclerotherapy with 0.2% povidone-iodine was conducted after 10 days, as the symptoms persisted. The patient had complete clearance of urine over the next five days. In case 2, the patient directly received sclerotherapy and had resolution of symptoms within three days of therapy.

Lymphatic fluids contain albumin and higher-molecular-weight globulins and fibrinogen, with a total protein concentration in the range of 3-6 g/dL [10]. A 24-h urine collection in patients with chyluria often reveals nephrotic-range proteinuria. This can lead to a misdiagnosis and a potentially unnecessary renal biopsy [6,7]. However, the absence of fatty cast cells and/or lipid-laden oval bodies, red blood cell and white blood cell casts in the urine, and hyperlipidemia differentiates chyluria from nephrotic syndrome.

Both of our patients had nephrotic-range proteinuria with hypoalbuminemia. Cheng et al. reported a similar case in which the patient presented with chyluria, heavy proteinuria, and pitting edema and was HCV positive, for which they conducted a renal biopsy to rule out HCV-related membranous or membranoproliferative glomerulonephritis, and minimal-change disease, but biopsy findings were unremarkable [2].

However, we deferred renal biopsy in both cases and directly managed the chyluria. The remission of proteinuria was achieved with the clearance of cloudy urine. Thus, it is important to obtain a detailed medical history, including the color and nature of urine, especially in patients with positive tests for urinary protein. Both cases were followed up for 6 months, with no recurrence of symptoms.

## Conclusions

Chyluria is a rare condition that can be mistaken for nephrotic syndrome because of its presentation with nephrotic-range proteinuria, hypoalbuminemia, and edema in individuals with malnutrition. However, normal or low lipid profile status should prompt examination of urine for chyle to prevent unnecessary invasive investigations and immunosuppressive treatment. The degree of proteinuria with chyluria is still undetermined and is under evaluation. Thus, further large-scale studies are needed.

## References

[1] Sinha RK, Ranjan N, Singh N, Amit K (2015). Chyluria: a scourge of our region. BMJ Case Rep.

[2] Sunder S, Jayaraman R, Mahapatra HS (2014). Analysis of case series of milky urine: a single center and departmental clinical experience with emphasis on management perspectives: a prospective observational study. Urol Ann.

[3] Graziani G, Cucchiari D, Verdesca S, Balzarini L, Montanelli A, Ponticelli C (2011). Chyluria associated with nephrotic-range proteinuria: pathophysiology, clinical picture and therapeutic options. Nephron Clin Pract.

[4] Diamond E, Schapira HE (1985). Chyluria - a review of the literature. Urology.

[5] Cheng JT, Mohan S, Nasr SH, D'Agati VD (2006). Chyluria presenting as milky urine and nephrotic-range proteinuria. Kidney Int.

[6] Dalela D (2005). Issues in etiology and diagnosis making of chyluria. Indian J Urol.

[7] Weil GJ, Ramzy RM (2007). Diagnostic tools for filariasis elimination programs. Trends Parasitol.

[8] Ohyama C, Saita H, Miyasato N (1979). Spontaneous remission of chyluria. J Urol.

[9] Hashim SA, Roholt HB, Babayan VK, Van Itallie TB (1964). Treatment of chyluria and chylothorax with medium-chain triglyceride. N Engl J Med.

[10] Dhabalia JV, Pujari NR, Kumar V, Punia MS, Gokhale AD, Nelivigi G (2010). Silver nitrate sclerotherapy for chyluria: evaluation for the optimal instillation regime. Urol Int.

[11] Kumar A, Suri A (2005). Chyluria-SGPGI experience. Indian J Urol.

[12] Abeygunasekera AM, Sutharshan K, Balagobi B (2017). New developments in chyluria after global programs to eliminate lymphatic filariasis. Int J Urol.

[13] Saha M, Ray S, Goswami M (2012). An occult filarial infection presenting as chyluria with proteinuria: a case report and review of literature. BMJ Case Rep.

[14] Sivashankar M, Nandasena AC (2020). A patient with milky urine: nonparasitic chyluria and silver nitrate sclerotherapy. Case Rep Urol.

